# Integrated pain management in a child with arthritis in Cape Verde: a case report

**DOI:** 10.3389/fped.2026.1811923

**Published:** 2026-07-08

**Authors:** Deisa Salyse dos Reis Cabral Semedo, Sandra de Sousa Lobo, Neusa Alfreda Mendonça Soares de Carvalho, Celestina Barros Martins, Elisabete Moreno Barros, Denise Castro Cardoso, Diva Elci dos Reis Cabral

**Affiliations:** 1 One Health Research Center, University of Cape Verde, Praia, Cabo Verde; 2 Faculty of Sciences and Technology, University of Cape Verde, Praia, Cabo Verde; 3 Department of Paediatrics, Dr. Agostinho Neto University Hospital, Praia, Cabo Verde

**Keywords:** Cape Verde, case report, integrated care, low-resource setting, paediatric pain

## Abstract

**Introduction:**

Juvenile idiopathic arthritis (JIA) is the most common chronic rheumatic disease in childhood, often associated with persistent pain, functional disability, and a negative impact on quality of life. Pain management is challenging, requiring multidisciplinary approaches that integrate biological, psychological, and social factors.

**Case presentation:**

This case report presents a clinical case of a 10-year-old male admitted for progressive polyarthritis with severe pain and an inability to walk. Laboratory and imaging tests confirmed enthesitis-related JIA (HLA-B27 positive). During a nine-month hospitalisation, pharmacological and non-pharmacological interventions were implemented, resulting in partial improvement and recurrent episodes of pain, leading to referral for biologic therapy at a specialized centre.

**Discussion:**

This case illustrates the complexity of chronic pain in JIA, with significant functional, emotional, and behavioural repercussions. Psychosocial factors and family involvement influence the experience of pain. Integrated, multidisciplinary, child-centred strategies that combine pharmacological and non-pharmacological interventions with emotional support are essential for effective management.

**Conclusion:**

In settings with limited resources, the lack of biologic therapies requires referral networks to ensure access to specialized care. This report highlights the importance of multidisciplinary, child-centred approaches to optimize the management of chronic pain in JIA.

## 1 Introduction

Juvenile idiopathic arthritis (JIA) is the most prevalent chronic rheumatic disease in children, with an estimated prevalence of approximately one in every 1,000 individuals. It affects children under 16 years of age, persists for more than six weeks, and represents a significant cause of short- and long-term disability (1, 2).

Children and adolescents with JIA experience lower health-related quality of life (HRQoL) compared to their healthy peers, due to the chronic and relapsing nature of the disease, its unpredictable course, the burden of symptom management, and the need for long-term treatment (1).

Pain is one of the most frequently reported experiences among children with JIA and is associated with multiple negative outcomes, including school absenteeism, reduced participation in activities, decreased quality of life, and mental health issues. Despite its high prevalence and substantial impact, pain management and control in JIA remain a significant clinical challenge. Previous studies have identified gaps in the training and self-efficacy of healthcare professionals in paediatric pain management, which, in some contexts, result in underestimating pain or omitting appropriate interventions in clinical practice (2, 3).

Beyond the biological factors inherent to the disease, psychosocial factors play a decisive role in the experience of pain in children. In the context of JIA, family dynamics, parental responses, and social support can significantly influence pain perception, disease adaptation, and the child's overall well-being, assuming particular relevance in situations of persistent pain and prolonged hospitalisation (3).

In this context, we present a clinical case report of a child diagnosed with enthesitis-related juvenile idiopathic arthritis who underwent prolonged hospitalisation (9 months). This case illustrates the main clinical and psychosocial challenges associated with the management of chronic pain in children, highlighting the role of healthcare teams in an integrated, multidisciplinary, and child-centred approach. The main objective of this work is to describe and evaluate how the paediatric healthcare team managed pain by promoting humanized, evidence-based care tailored to the child's individual needs.

## 2 Case presentation

### 2.1 Identification and general context

The patient is a 10-year-old male from the interior of Santiago Island, Cape Verde, under follow-up at the Paediatric Department of Dr. Agostinho Neto University Hospital, in the city of Praia. He was admitted with a history of pain, swelling, and functional impairment in both knees, which started approximately one month prior (April 2025). He reported that the symptoms began following a fall in which he struck his knees. He denied any previous complaints, including pain or swelling in other joints, fever, or skin, eye, or other lesions.

He has no history of other hospitalisations, allergies, or chronic illnesses. His developmental milestones are appropriate for age, and his national immunization schedule is up to date. School performance has been reported as good.

Regarding relevant family history, it is worth noting that the patient's older brother presented with a similar clinical picture during childhood; he was also admitted to the same hospital and currently has a diagnosis of ankylosing spondylitis for which he is receiving ongoing treatment in Portugal. The family also has a history of neurological disorders on the maternal side, specifically stroke and epilepsy.

Regarding the family and social context, the patient comes from a household consisting of the mother and five children. He lives only with four healthy siblings aged 19, 15, 5, and 3; the 23-year-old older brother resides in Portugal. The father has emigrated to Portugal and is absent from daily family life.

During the hospitalisation period, the mother and the older sister initially actively assisted the child with care, particularly regarding hygiene and feeding. However, as the hospitalisation progressed, their presence in the ward became less regular, and they began making only short visits.

This change was associated with the family's socioeconomic conditions. The presence of other minor children in their care and the geographical distance between the family residence and the hospital made it difficult to make regular visits. Later, the family moved to the city of Praia to facilitate hospital visits. Nevertheless, financial constraints and a limited support network continued to pose obstacles to frequent visits.

### 2.2 Clinical examination

On physical examination, the child was in fair general condition, alert and oriented, well-hydrated, breathing normally, and had a normal skin colour. The axillary temperature was 37.2 °C and body weight was 22.2 kg. The oropharynx showed no lesions. Cardiac auscultation revealed normal, rhythmic heart sounds without murmurs. Pulmonary auscultation revealed preserved bilateral vesicular breath sounds, without rales or stridor. The abdomen was flat, soft, and painless, with no organomegaly.

The musculoskeletal examination revealed swelling in both knees with local warmth, pain, stiffness, and mild erythema, along with limited active and passive range of motion due to pain and an inability to walk. Both ankles were painless on palpation but exhibited local warmth. In the right hand, oedema, pain, and warmth were observed in the proximal interphalangeal joints of the third and fourth fingers and in the interphalangeal joint of the first finger. In the left hand, there was pain in all proximal interphalangeal joints, metacarpophalangeal joints, and in the interphalangeal joint of the first finger. Functional mobility was not assessed.

### 2.3 Complementary investigations

Laboratory and imaging tests were carried out as part of the diagnostic assessment and to monitor clinical progression. The main results are shown in Table 1.

**Table 1:** Laboratory investigations during hospitalisation. Reference ranges are shown in parentheses.

Laboratory findings	Reference values	13 May 2025	30 May 2025	25 July 2025	24 September 2025	11 October 2025	1 December 2025	3 February 2026
Haemoglobin (Hb), g/dL	11–14	10.1	9.4		9.3	8.6	7.7	7.3
Haematocrit (Hct), %		32	29		30.3	28.6	26.4	25.1
Mean corpuscular volume (MCV), fL	79–90	77	74		71.3	69	68	60
White blood cell count (WBC), cells/mm³	4.5–14.500	12,300	10,800		17,000	12,500	16,000	10,900
Neutrophils/Lymphocytes/Monocytes/Eosinophils (%)		43/43/10/4	57/28/12/		50/36/12	82/9.5/7.3	54/33/11/0.7	49/34/14/1.4
Platelet count, cells/mm³	150–400	4,92,000	6,42,000		8,14,000	7,74,000	5,53,000	7,10,000
Erythrocyte sedimentation rate (ESR), mm/h	2–15	77		25	107		67	78
Antistreptolysin O titre (ASO)		714		299				
C-reactive protein (CRP), mg/L	≤1	6,67						
Complement C3, g/L	0,93–1.18	1.4	2,11					
Complement C4, g/L	0,15–0,48		0,41					
Thyroid-stimulating hormone (TSH), µIU/mL	0.25–5.00		1,879					
Serum iron, µg/dL			10					
Ferritin, ng/mL	16–243		249					
Reticulocyte count, %			1.34					
Blood glucose, mg/dL				85		117		71
Urea, mg/dL	17–43				12		22	13
Creatinine, mg/dL					0.47	0,43	0.47	0,45
Aspartate aminotransferase (AST), U/L				20	29		21	17
Alanine aminotransferase (ALT), U/L	≤45			17			20	7
Sodium/Potassium (Na/K), mEq/L	136–146/3.5–5.6					136/4,7/99	135/4.4/96	132/4.6/99
Gamma-glutamyl transferase (GGT), U/L							42	
Calcium, mg/dL						9,19	9.63	9.1
Phosphate/Magnesium, mg/dL						4,4	5.8/1.88	5.6/1.83
Total protein/Albumin, g/dL			8,2/3,58					
Lactate dehydrogenase (LDH), U/L			202					161
Triglycerides, mg/dL			62					
Total cholesterol/HDL/LDL cholesterol, mg/dL			138/32/32					
Glycated haemoglobin (HbA1c), %				5.4				
Vitamin B12, pg/mL			368					
Folate, ng/mL			9,86					

#### 2.3.1 Laboratory

- 2 June 2025–24-hour urinary protein: 0.07 g/24 h; no sickle erythrocytes detected.
- 11 June 2025—Anti-double-stranded DNA antibodies (Anti-dsDNA) < 48 U/mL; anti-SSA (Ro), anti-SSB (La) antibodies, anti-ribonucleoprotein antibodies (anti-RNP), anti-smooth muscle antibodies (SMA), anti-cyclic citrullinated peptide (anti-CCP) IgG antibodies and antiphospholipid antibodies (IgG 1.6 GPL-U/mL and IgM 5.2 MPL-U/mL)—negative.
- 14 July 2025—Human immunodeficiency virus (HIV), hepatitis B surface antigen (HBsAg) and hepatitis C virus (HCV)—negative.
- 12 August 2025—Antinuclear antibodies (ANA) and cytoplasmic antibodies—negative.
- HLA-B27 genotyping—positive.

#### 2.3.2 Imaging

- Ultrasound of the soft tissues of the wrists: mild signs of synovitis and soft tissue oedema, with no evidence of fluid collections.
- 2 December 2025—Computed tomography scan of the sacroiliac joints: preserved joint spaces; intact soft tissues; no evidence of inflammatory changes in bone or soft tissues.

The patient presented with mild anaemia on admission, associated with his chronic condition, and this progressively worsened as the disease progressed. This culminated in the need for a blood transfusion to ensure he was fit for the evacuation. It is also worth noting the increase in inflammatory markers such as erythrocyte sedimentation rate (ESR), which fluctuated in line with exacerbations of the condition. Liver and kidney function remained normal throughout.

### 2.4 Diagnostic assessment

#### 2.4.1 28 may 2025—admission

The child was admitted due to persistent signs of inflammation affecting the knees, ankles, wrists and proximal interphalangeal joints of the hands.

In the context of polyarthritis of uncertain aetiology, several diagnostic hypotheses were considered, including juvenile idiopathic arthritis, rheumatic fever, infectious vs. inflammatory arthritis, and sickle cell anaemia.

Anti-inflammatory treatment was initiated. The child was initially treated with acetylsalicylic acid (ASA), followed by ibuprofen and metamizole, with an unsatisfactory clinical response. Due to the possibility of arthritis associated with rheumatic fever, a single intramuscular (IM) dose of 1,200,000 international units (IU) of benzathine penicillin was also administered.

The child was assessed by Cardiology, which ruled out heart disease or valvular disease, and by Orthopaedics, which ruled out orthopaedic pathology, and a conservative management approach was maintained. Assessment by Rheumatology confirmed polyarthritis of undetermined aetiology, with juvenile idiopathic arthritis considered a possible diagnosis.

### 2.5 Therapeutic intervention and clinical course

Throughout the hospital stay, an integrated approach was implemented, combining pharmacological and non-pharmacological interventions, with the involvement of a multidisciplinary team comprising nursing, medicine, psychology, physiotherapy and social work, as described below. Pharmacological treatment included various analgesic and anti-inflammatory regimens, progressively adjusted according to clinical response (Tables 2, 3). Of note was the use of non-steroidal anti-inflammatory drugs, corticosteroids, opioids and disease-modifying drugs, which provided an overall improvement in pain, although intermittent episodes of exacerbation and joint stiffness persisted.

**Table 2:** Medications used for pain and inflammation (analgesics, opioids, corticosteroids, and NSAIDs).

Administration period	Medication	Dose	Pre-intervention pain score	Post-intervention pain score	Clinical Response
28 May–03 June 2025	Ibuprofen/Metamizole (oral)	200 mg every 8 h/250 mg every 6 h (as needed)	8/10	3/10	Transient improvement
24 June 2025	Metamizole (IV^2^)	300 mg every 8 h, as needed	8/10	2/10	Transient improvement
05 June–03 July 2025	Tramadol (IV)	25 mg every 12 h	8/10	2/10	Transient improvement
10 October 2025–27 November 2025	Paracetamol (oral)	400 mg every 8 h	8/10	3/10	Transient improvement
03 June 2025–26 January 2026	Prednisolone (oral)	30 mg/day (1,5 mg/kg/day)	8/10	3/10	Initial improvement; Gradual taper: 1 mg/kg/day → 0.5 mg/kg/day
1st cycle	Methylprednisolone (IV), 3 cycles of 3 pulses	400 mg every 12 h	--	--	Transient improvement
13–15 June 2025					
2nd cycle					
22–24 July 2025					
3rd cycle					
25–26 September 2025					
11 September 2025–18 December 2025	Naproxen (oral)	250 mg every 12 h	8/10	3/10	Transient improvement
19 August 2025–18 December 2025	Sulfasalazine (oral)	10/mg/day. Gradual weekly titration up to 50 mg/kg/day			Initial improvement, later stabilization with intermittent pain and stiffness.
10 October 2025–1 December 2025	Morphine (oral)	5 mg every 4 h	8/10	2/10	5 mg dose maintained, with gradually increasing dosing intervals (6-hourly → once daily) and subsequent discontinuation. As-needed dose: 5 mg.

**Table 3:** Supportive medications.

Administration period	Medication	Dose	Clinical Response
15 July 2025	Methotrexate	2,5 mg weekly (for 5 weeks)	Initial improvement
3 July 2025–18 December 2025	Omeprazole (oral)	20 mg/day	
13–17 June 2025	Albendazole	400 mg/day	

All pain intensity ratings reported in this case were obtained using the Numeric Rating Scale (NRS), ensuring a standardized and systematic assessment of pain throughout the clinical course, in line with validated paediatric pain assessment tools (4).

Non-pharmacological interventions were also implemented, including continuous motor physiotherapy, encouragement to participate in adapted recreational activities, and emotional support strategies, led by a multidisciplinary team comprising physiotherapists, a psychologist and a nursery teacher. The latter is a professional who is usually present in the paediatric ward, ensuring the continuity of learning and the emotional well-being of hospitalised children. They use play-based activities, toy libraries and individualised lessons to minimise the impact of hospitalisation, adapting the school curriculum and fostering a welcoming environment, thereby humanising medical care.

These interventions were carried out throughout the entire hospital stay, with frequency and approach adjusted to the child's clinical condition and needs at different stages of their recovery, contributing to their physical and psychological well-being.

#### 2.5.1 3 june 2025—initiation of steroid treatment

Due to persistent severe polyarticular pain associated with significant functional impairment, intravenous prednisolone was initiated. Subsequently, the first pulse of methylprednisolone was administered on 13, 14 and 15 June 2025, with a favourable clinical response, characterised by a reduction in pain from NRS score of 7–8/10 to 2–3/10 and a decrease in inflammatory signs. Oral prednisolone was continued at a dose of 1 mg/kg/day; however, subsequent worsening of pain and inflammatory signs was observed, which led to the administration of a second pulse of methylprednisolone on 22, 23 and 24 July 2025, and a third pulse on 25 and 26 September 2025. Each pulse resulted in a good clinical response, although the effect was transient.

#### 2.5.2 25 july 2025

Methotrexate was initiated at a weekly dose of 2.5 mg, with a favourable clinical and laboratory response, evidenced by a reduction in erythrocyte sedimentation rate (ESR). With physiotherapy support, the patient achieved partial recovery of ambulation, presenting periods of pain remission, although joint stiffness persisted in the knees and wrists.

#### 2.5.3 August 2025—clinical deterioration

Clinical deterioration was observed, with involvement of multiple joints, including the temporomandibular joint (with trismus) and the right sacroiliac joint, associated with pain at rest. Given the diagnosis of enthesitis-related arthritis (ERA), with HLA-B27 positivity, methotrexate was discontinued and sulfasalazine was initiated on 19 August 2025, initially at a dose of 10 mg/kg/day, with weekly dose escalation up to 50 mg/kg/day, which was maintained until the date of transfer (9 February 2026). Initial clinical improvement was observed.

#### 2.5.4 October 2025—periods of exacerbation

Worsening of the clinical condition occurred, characterised by episodes of severe joint pain and inability to walk. The child reported moderate to severe pain (6–9/10), associated with joint stiffness involving the knees, wrists, temporomandibular joints, and right sacroiliac joint. During this period, episodes of trismus and food refusal were also noted.

Due to worsening pain, analgesic therapy was initiated on 10 October 2025 with oral morphine 5 mg every 4 h and paracetamol 400 mg every 6 h; paracetamol was maintained until 27 November 2025, with progressive improvement in pain complaints. Despite the instituted therapy, during exacerbations the patient reported pain intensity up to 8/10, with persistent joint stiffness and significant limitation of activities of daily living and leisure activities.

Given refractoriness to ongoing therapy and the indication for initiation of biological therapy, which was unavailable locally, the case was presented to the Sotavento Medical Board, and medical evacuation abroad was proposed for evaluation and follow-up at a specialised paediatric rheumatology centre.

#### 2.5.5 November 2025 to february 2026—progressive improvement until transfer

Following the period of greatest symptom severity, progressive clinical improvement was observed. The child presented only occasional episodes of pain (2–3/10) and joint stiffness, predominantly in the morning. On most days, he appeared well, participated in recreational activities, ambulated within the ward, and attended the activity room. When clinical conditions did not allow movement to leisure areas, activities were carried out in the hospital room with support from the early childhood educator.

Despite clinical improvement, joint stiffness and deformities persisted until the date of transfer, particularly affecting the wrists and knees.

During this period, the morphine dose was progressively reduced and discontinued on 1 December 2025, thereafter, maintained only on an as-needed basis during painful exacerbations.

Throughout hospitalisation, blood pressure and blood glucose values remained within normal limits. The patient weighed 22 kg. In the final laboratory tests performed prior to travel, moderate anaemia was identified, and a packed red blood cell transfusion was administered on 9 February 2026.

## 3 Discussion

Chronic pain is one of the most frequent and impactful manifestations of juvenile idiopathic arthritis (JIA), and it may persist beyond the objective inflammatory activity of the disease (1). This case describes a child with JIA associated with enthesitis, prolonged hospitalisation, and recurrent episodes of moderate-to-severe pain, with significant functional, emotional, and behavioural consequences, illustrating the complexity of pain management in this population.

This finding is consistent with evidence that chronic musculoskeletal pain is highly prevalent in children. Chambers et al. (5) estimate that approximately 1 in 5 children and adolescents experience chronic pain, with musculoskeletal pain being among the most frequent presentations. Therefore, the persistence of pain observed in this case, despite the instituted therapeutic approach, should not be interpreted as an exceptional event, but rather as part of a phenomenon widely described in the paediatric literature, requiring systematic assessment and intervention strategies.

Beyond the biological factors inherent to JIA, paediatric pain should be understood as a multidimensional experience influenced by emotional, behavioural, and contextual factors. Brandelli et al. (3) demonstrate that psychosocial variables, such as mood, coping strategies, family functioning, and parental perception of pain, are strongly associated with pain intensity and its interference with daily life in children with JIA. These findings underscore the importance of clinical approaches that go beyond a purely biomedical model.

In the present case, the child exhibited marked mood fluctuations, irritability, and behavioural changes. The family had difficulty recognizing these manifestations as possible expressions of pain, which contributed to recurring episodes of conflict between the boy, his mother, and his sister.

The literature indicates that psychosocial factors, including parental responses to pain and family coping styles, significantly influence the intensity and persistence of pain in children with JIA. Adequate parental involvement is recognized as a central element in regulating paediatric pain and promoting adaptive coping strategies, while the absence of structured caregiver education strategies may contribute to misinterpretation of pain and dysfunctional family responses (3, 6).

The relatively limited family involvement observed during hospitalisation appeared to be associated primarily with socioeconomic and logistical constraints, rather than a lack of engagement. Nevertheless, the reduced presence of caregivers may influence the child's painful experience, as parental involvement is recognized as an important factor in the modulation of paediatric pain and the effectiveness of interventions.

Chronic pain associated with JIA has significant implications for health-related quality of life, affecting emotional well-being, social participation, and school performance. Richmond et al. (7) show that children and adolescents with JIA experience worse psychosocial outcomes when pain is persistent, independent of disease severity. Complementarily, Spillane e Murray (2) highlights that pain, fatigue, and emotional instability are determinants of reduced school attendance and participation in daily activities.

Beyond immediate impact, chronic pain in childhood is associated with significant long-term functional impairment. Children experiencing chronic pain are at increased risk of developing depression and anxiety, social isolation, school absenteeism, and lower quality of life, with pain frequently persisting into adulthood. This phenomenon represents a substantial source of stress not only for the child but also for the family and healthcare systems (5), reinforcing the importance of early and adequate intervention.

In the present case, prolonged hospitalisation and functional limitations contributed to withdrawal from school and social contexts, negatively impacting mood and emotional adaptation. The observed emotional and behavioural suffering might be interpreted as the result of the interaction between persistent pain, hospitalisation, and disruption of usual routines, as described in recent literature (2, 7).

The management of paediatric chronic pain requires an integrated approach combining pharmacological and non-pharmacological strategies. In this context, the World Health Organization (WHO) published, in 2020, specific guidelines for the management of primary and secondary chronic pain in children and adolescents aged 0–19 years, recommending physical, psychological, and pharmacological interventions based on the best available evidence (8). These guidelines emphasize the need for structured, multidisciplinary, and child-centred care.

The review by Checa-Peñalver et al. (9) demonstrates that non-pharmacological interventions, including cognitive, behavioural, play-based, and emotional support strategies, are associated with reduced pain intensity and improved overall well-being. In the context of JIA, interventions promoting adapted physical activity have also shown benefits, with structured programs considered safe and effective in improving function and reducing perceived pain (10).

Studies have also highlighted the importance of structured support programs for children with JIA that go beyond pharmacological therapy, incorporating educational support, continuous communication, and psychosocial follow-up (11).

In a qualitative study involving children enrolled in a support program that provided regular visits and ongoing contact during the first year after diagnosis, participants reported that the integration of clinical guidance, accessible information, and emotional support contributed to an overall sense of safety and well-being throughout the course of treatment. These findings suggest that structured, child and family-centred approaches may enhance the care experience and promote greater equity in health outcomes among young people with JIA (11).

The intervention implemented by the medical and nursing team in the present case, including systematic pain assessment (NRS), encouragement of mobility, play activities, and emotional support, aligns with current recommendations. However, during episodes of intense pain, other validated paediatric pain assessment scales, such as the Face, Legs, Activity, Cry, Consolability (FLACC) scale or the Wong-Baker FACES scale, could have been considered.

Treatment adherence represents a central challenge in children with chronic diseases. Nelson et al. (6) emphasize that emotional factors, parental beliefs, and the quality of communication with healthcare professionals directly influence therapeutic adherence in youth with JIA.

In the present case, the family's difficulty in understanding the fluctuating nature of pain and associated behavioural manifestations may have affected the effectiveness of the therapeutic plan, underscoring the need for targeted caregiver education and structured communication strategies.

This report has inherent limitations due to its descriptive nature and focus on a single case, preventing the generalisation of results. The absence of standardised psychosocial assessment tools and limited complementary objective data, such as detailed laboratory tests, also constrains in-depth analysis of clinical evolution.

On the other hand, the described case highlights that caring for a child with chronic rheumatic disease during a prolonged hospital stay requires an integrated approach that goes beyond pharmacological pain management, encompassing clinical, functional, emotional, and family dimensions. The coordinated mobilization of available professionals, including physicians, nurses, physical therapists, psychologists, and a social worker, made it possible to implement a multidisciplinary approach tailored to the needs of the child and the family.

Despite the partial response to the treatments administered, the need for biologic therapy, which was not available locally, led to the child's referral to a centre with a broader range of treatment options, highlighting the importance of collaborative and referral networks in ensuring access to specialized treatments when necessary.

## 4 Conclusion

This case report highlights the complexity of managing chronic pain in children with juvenile idiopathic arthritis, particularly in the context of prolonged hospitalisation and limited family involvement.

It underscores the importance of integrated, multidisciplinary, and child-centred approaches, supported by systematic pain assessment and careful attention to psychosocial and cultural factors that influence disease experience. Strengthening structured clinical protocols, investing in continuous training of healthcare professionals, and optimizing available resources are key strategies for improving paediatric pain management and child health outcomes, particularly in resource-limited settings such as Cape Verde.

Dr. Agostinho Neto University Hospital maintains institutional collaboration with Portugal's National Health System, under an existing bilateral cooperation agreement with the Government of Cape Verde, which allows for patient transfer when clinical conditions exceed the therapeutic resources available locally. In this context, the child was evacuated to Portugal on February 9, 2026, as it was determined that his condition was refractory to the therapy available within the local healthcare system.

This case highlights the challenges of managing complex paediatric rheumatological conditions in settings where certain highly specialized therapeutic resources are not available. Despite the commitment of the multidisciplinary team during hospitalisation, referral to a specialised centre was required due to the absence of biologic therapy, to ensure continuation of care. It is expected that specialized care in Portugal will lead to a favourable clinical outcome and eventual reintegration of the child into the family and community.

## Data Availability

The original contributions presented in the study are included in the article/Supplementary Material, further inquiries can be directed to the corresponding author.

## Footnotes

^2^Intravenous
